# Ethylene Glycol Toxicity in the Setting of Recurrent Ingestion: A Case Report and Literature Review

**DOI:** 10.7759/cureus.4375

**Published:** 2019-04-03

**Authors:** Daniel Loriaux, Stephen P Bergin, Sweta M Patel, Jesse Tucker, Christina E Barkauskas

**Affiliations:** 1 Internal Medicine, Brigham & Women’s Hospital, Harvard Medical School, Boston, USA; 2 Internal Medicine - Critical Care, Duke University Medical Center, Durham, USA

**Keywords:** ethylene glycol toxicity, osmolal gap

## Abstract

Ethylene glycol (EG) poisoning is a toxicologic emergency requiring high clinical suspicion and early diagnosis to prevent life-threatening complications. Direct EG quantification methods involve cumbersome and time-consuming laboratory tests of limited utility in the emergency setting. Accordingly, the osmolal gap is frequently employed as a surrogate screening method in cases of suspected toxic alcohol poisoning. However, the osmolal gap has several inherent limitations to be considered when used as a diagnostic tool for EG toxicity. Although many of these limitations are widely acknowledged, the clinical finding of a normal serum osmolal gap in the setting of recurrent toxic alcohol exposure is an observation that has remained largely unexplored. The purpose of this case report is to characterize the accelerated osmolal gap to anion gap conversion that may occur in the setting of chronic toxic alcohol abuse.

## Introduction

Ethylene glycol (EG) is an odorless, electrically neutral polyalcohol and a common solvent for antifreeze solutions, brake fluids, and household industrial products [1-2]. The sweet taste, low cost, and intoxicating effects of EG lead to its illicit use as an ethanol substitute [2-3]. In cases of both accidental and purposeful ingestion, timely diagnosis of EG toxicity enables the administration of agents that block the formation of toxic metabolites (ethanol or fomepizole) or initiation of hemodialysis to avoid systemic, irreversible, and life-threatening complications [4-6]. However, early diagnosis and intervention is challenging for several reasons. The initial clinical presentation is nonspecific, involving a constellation of symptoms including altered sensorium, headache, slurred speech, ataxia, nystagmus, nausea, and vomiting. When EG ingestion is suspected, direct serum assays are limited in availability and many institutions must resort to send-out laboratory testing [7]. When direct detection methods are available, the prolonged turnaround time to perform the necessary diagnostic studies precludes direct screening in the emergency setting [8]. The osmolal gap is a substitute indicator widely regarded as the best available screening test for toxic alcohol ingestion [9-10].

Although commonly utilized, the osmolal gap lacks sensitivity. This is due to the fact that EG (an uncharged, osmotically active parent alcohol) is oxidized by alcohol dehydrogenase (ADH) into toxic metabolites that are negatively charged at physiological pH.

These metabolites exist in a dissociated state and are accompanied by a cation (sodium), which makes them undetectable in the osmolal gap [6]. Diagnostically this manifests as the disappearance of the osmolal gap in exchange for a rising serum anion gap and an artefactual lactic acidosis, which is due to the cross-reaction of the EG metabolites with L-lactate oxidase in blood gas analyzers and lactate electrodes [11].

During EG metabolism, the transition point between the elevated osmolal gap and anion gap is largely dependent on the enzymatic activity of ADH, which is influenced by age, gender, body mass, nutrition, exercise, and prior exposure to alcohol [12]. Similar to the enhanced ADH activity that is observed in chronic alcoholism, in vivo research shows that the efficiency of ADH in metabolizing toxic alcohols is amplified by chronic exposure [13]. This is clinically significant because the increased metabolic efficiency of ADH in the setting of chronic toxic alcohol abuse may significantly shorten the duration an elevated osmolal gap remains detectable. The case presented in this report highlights the clinical importance of accelerated EG metabolism and the potentially misleading nature of lactic acidosis when both the osmolal gap and toxicology studies are found to be normal.

## Case presentation

A 38-year-old woman with a past medical history of chronic alcohol abuse, seizures, and recurrent hospitalizations for profound lactic acidosis of unknown etiology presented to the Emergency Department (ED) with altered sensorium and shortness of breath. The patient had been discharged from the hospital 12 hours earlier after management of a similar illness. The current presentation was her sixth hospital admission within the previous six months. All prior presentations shared similar symptoms and laboratory findings: acute onset altered mental status, slurred speech, unsteady gait, tachycardia, and tachypnea in the setting of leukocytosis, acute kidney injury, profound lactic acidosis (ranging from 10-30 mmol/L), high anion gap (often greater than 30 mEq/L), normal osmolal gap (5-10 mOsm/kg), and negative toxicology studies. Although EG ingestion had been considered during many of these prior admissions, the patient’s uniformly low osmolal gap, normal urinalysis, marked lactic acidosis, and negative blood volatile studies had prompted broadening of the differential and extensive evaluation for a suspected mitochondrial, infectious, or inherited metabolic disorder. Despite repeated and exhaustive evaluations, no definitive toxic, infectious, or metabolic etiology had been identified.

On arrival to the ED, the patient was afebrile (36.3 °C), tachycardic (114 beats per minute), and tachypneic (22 breaths per minute) with an oxygen saturation of 100% while breathing ambient air. Her initial laboratory studies were remarkable for a white blood cell count of 18.9 K/uL (ref: 4-10.9 K/uL), pH 7.13 (ref: 7.35-7.45), anion gap 35 mEq/L (ref: 3-15 mEq/L), lactic acid 14 mmol/L (ref: 0.5-2.0 mmol/L), osmolal gap 5 mOsm/L (ref: <10 mOsm/L), normal chemical and microscopic urinalysis, negative serum and urine toxicology studies, and undetectable blood volatile organic compounds. Despite the absence of an osmolal gap, her anion gap and lactic acidosis prompted concern for EG ingestion and fomepizole therapy was prophylactically initiated.

**Figure 1:**
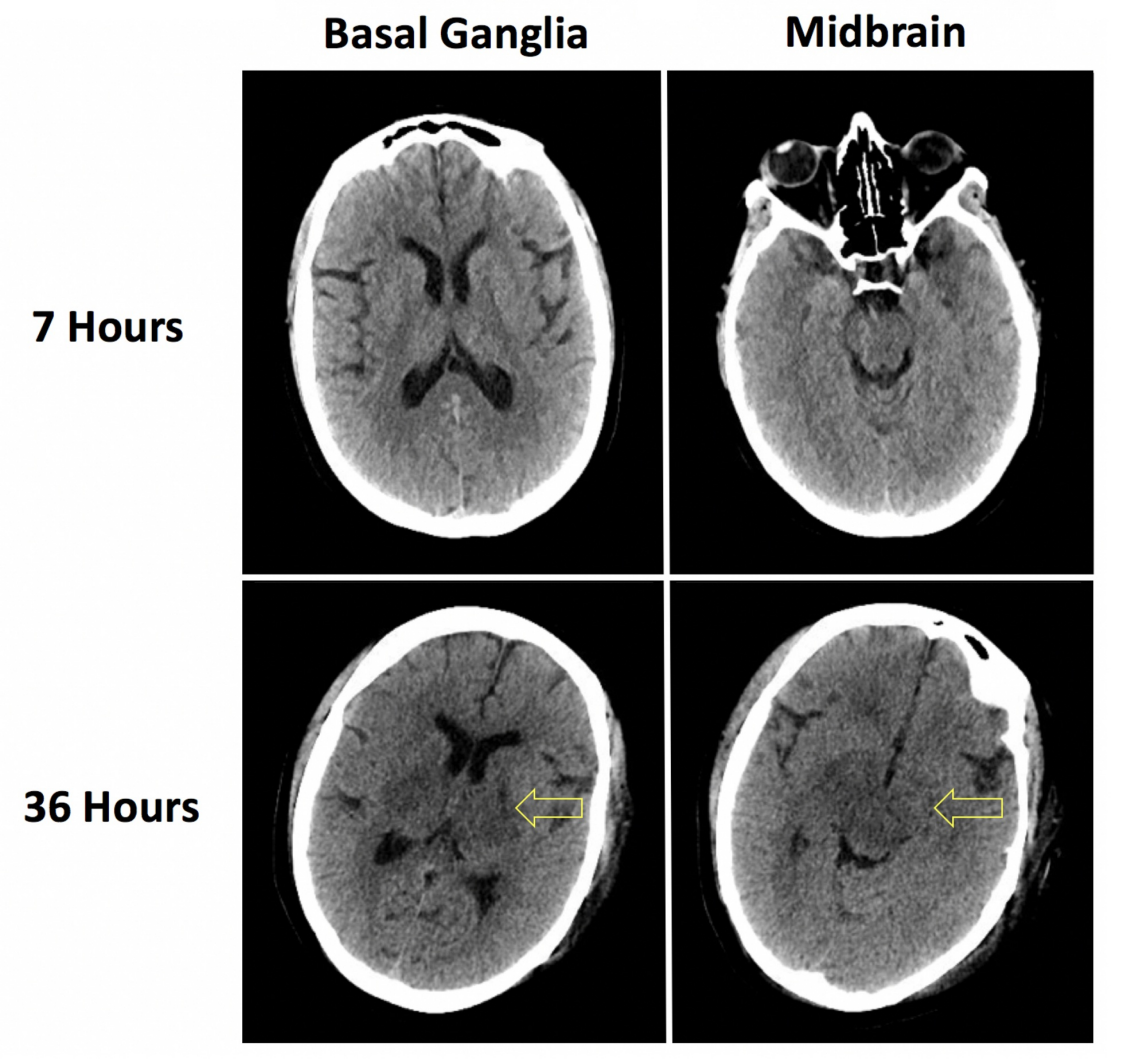
Noncontrast computed tomography imaging Noncontrast head computed tomography imaging acquired 7 hours and 36 hours after presentation revealing interval development of basal ganglia and midbrain hypoattenuation. Although nonspecific, these radiographic findings are consistent with ethylene glycol toxicity.

Three hours after her arrival to the ED (15 hours after her most recent hospital discharge), the patient declined with a rapid deterioration in mental status accompanied by acute hypoxemic respiratory failure. She was intubated and admitted to the medical intensive care unit. The first 24 hours of her hospital course were complicated by refractory shock, complete heart block, new non-ST elevation myocardial infarction accompanied by severe systolic heart failure with an estimated left ventricular ejection fraction of 15%, and persistence of the severe anion gap metabolic acidosis (30 mEq/L). Hemodialysis was initiated. On hospital day 2, she became febrile to 40.3 °C with worsening hyperkalemia and hyperphosphatemia despite hemodialysis. The neurologic examination declined from Glasgow Coma Scale (GCS) 15 with no focal neurologic deficits on admission to GCS 3 with absent brainstem reflexes. Repeat noncontrast head computed tomography acquired 36 hours after presentation revealed interval development of bilateral basal ganglia and midbrain hypoattenuation, a nonspecific radiographic finding that can arise secondary to infectious etiologies, toxic insults, inflammatory conditions, or metabolic disorders (Figure 1) [14].

In accordance with the family’s wishes, the supportive care was withdrawn 72 hours after the admission. All the final culture results and toxicology studies returned negative. At the request of the family, an autopsy was performed and the case was referred to the medical examiner.

**Figure 2:**
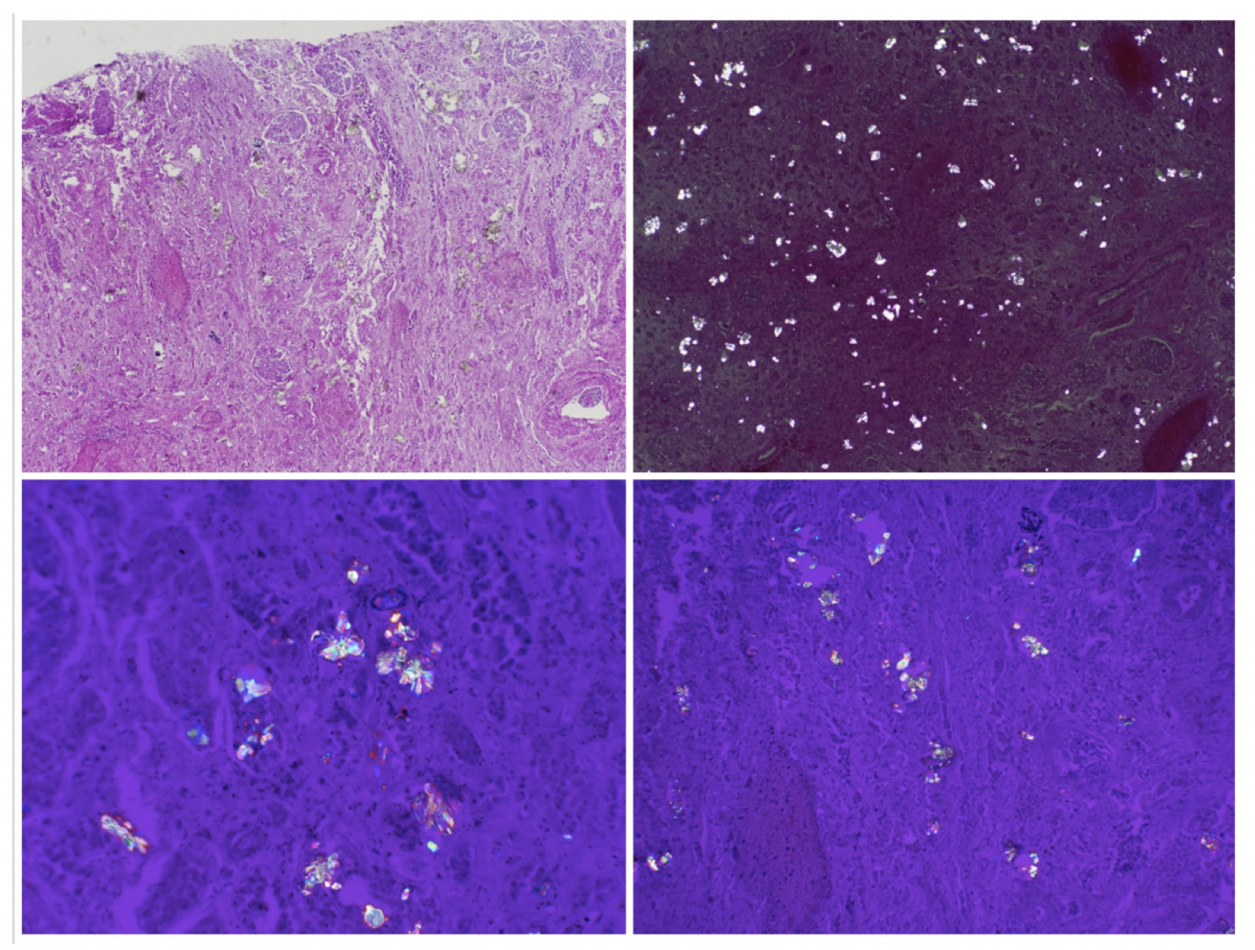
Autopsy findings Photomicrographs showing diffuse calcium oxalate deposition throughout the renal parenchyma, a finding pathognomonic for ethylene glycol poisoning.

Autopsy revealed extensive intravascular and perivascular oxalate crystal deposition. Intravascular foreign material, consistent with oxalate crystals, was identified throughout the renal parenchyma and brain. These findings are pathognomonic for EG ingestion (Figure 2). 

## Discussion

**Figure 3:**
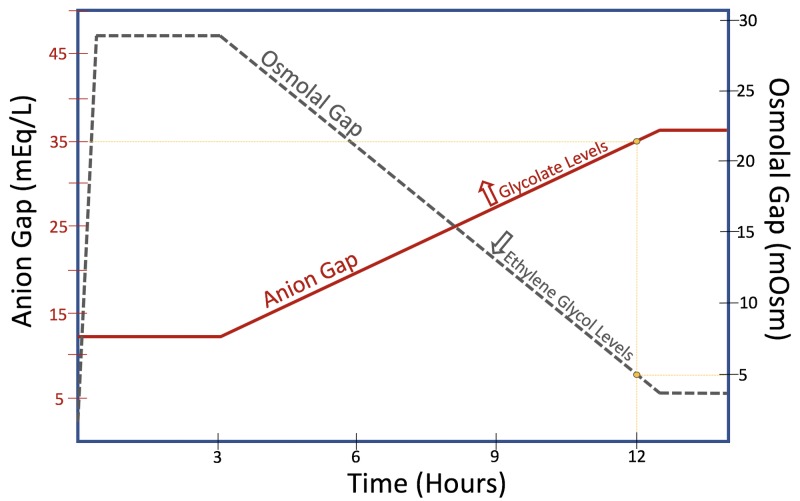
Osmolal gap to anion gap conversion Visual schematic depicting the relationship between the osmolal gap (solid line) and anion gap. (dashed line). As ethylene glycol is metabolized into toxic, non-osmotic metabolites (i.e. glycolate), the osmolal gap decreases and the anion gap begins to rise.

EG toxicity characteristically presents as a severe metabolic acidosis with a high anion gap (27.8 +/- 12.4 mEq/L), elevated osmolal gap (33.3 +/- 5.99 mOsm/L) and large mean base deficit (18.6 +/- 10.9 mEq/L) [15]. A significantly elevated osmolal gap is highly specific but poorly sensitive for EG poisoning [16-17]. Pure EG is rapidly metabolized and the likelihood of detecting an osmolal gap decreases with time following ingestion (Figure 3). It has been demonstrated in vivo that the enzymatic efficiency of ADH in metabolizing EG monohydrate and eliminating the osmolal gap may be accelerated in the setting of multiple past toxic alcohol exposures [13]. The present case is unique because of the documented 12-hour interval between hospital discharge and readmission; the patient’s clinical history suggests that it was only several hours prior to collection of admission laboratory studies that lethal ingestion of EG occurred. The absence of an osmolal gap in this case supports the concept that the efficiency of toxic alcohol metabolism can be significantly enhanced in patients with past exposures.

Artefactual lactic acidosis is an additional complicating factor in the diagnosis of EG poisoning. Glycolate, one of the primary metabolites of EG, is structurally similar to lactate and high circulating levels of glycolate following the EG consumption can be falsely interpreted as a lactic acidosis [11]. Additionally, glycolate is a tasteless and odorless powder that is marketed over the counter as a primary ingredient in many cosmetic products and skin emollients. A toxic ingestion of glycolate would be undetectable in the osmolal gap and virtually indistinguishable from a delayed presentation of the EG poisoning. An understanding of the association between glycolate, artefactual lactate and EG is critical for formulating the complete differential and reaching an early and accurate diagnosis.

The case described in this report is unique in that a lethal EG exposure with a normal osmolal gap confirmed within 12 hours of the toxic ingestion has not been previously reported. A normal osmolal gap has been found only in a small subset of patients suffering from severe EG intoxication, and all of these cases involve patients who presented more than 12 hours following ingestion (Table 1) [2, 18-19].

**Table 1:** Reported cases of ethylene glycol toxicity with a normal osmolal gap

Patient	Anion Gap (mEq/L)	Osmolal Gap (mOsm)	Time: Consumption to Presentation
71yo Male [19]	40	4	>24 hours
35yo Male [19]	48	7	>24 hours
42yo Male [6]	42	9	48 hours
49yo Male [20]	32	4	10 days
23yo Male [18]	25	7.2	12-24 hours

Notably, the only documented case of EG exposure with a normal osmolal gap presenting close to 12 hours from the EG ingestion involved a patient who similarly had a history of prior toxic alcohol consumption [18]. 

## Conclusions

For patients with a history of toxic alcohol abuse, the duration of time required to convert EG into its non-osmotically active toxic metabolites can be significantly decreased. In this subset of patients, the osmolal gap will be within normal limits shortly after ingestion and an accelerated progression through the stages of EG toxicity can occur. Severe lactic acidosis in the setting of a normal osmolal gap in a chronic alcoholic patient should be interpreted with caution, as this can be found in patients with chronic EG exposure.
